# Remote Cerebellar Hemorrhage Following Lumboperitoneal Shunt Insertion: A Rare Case Report

**DOI:** 10.1055/s-0036-1594245

**Published:** 2016-12-01

**Authors:** Fatih Ayvalık, Rafet Ozay, Erhan Turkoglu, Mehmet Serdar Balkan, Zeki Şekerci

**Affiliations:** 1 Clinic of Neurosurgery, Ministry of Health Dışkapı Yıldırım Beyazıt Training and Research Hospital, Ankara, Turkey

**Keywords:** cerebellar hemorrhage, complication, lumboperitoneal shunt

## Abstract

Idiopathic intracranial hypertension is characterized by high intracranial pressure without hydrocephalus or intracranial mass. Surgical treatment includes optic nerve fenestration and insertion of ventriculoperitoneal and lumboperitoneal (LP) shunts. For decreasing intracranial pressure, cerebrospinal fluid (CSF) LP shunt is widely used for the surgical management; it also carries complications such as shunt migration, venous sinus thrombosis, subarachnoid hemorrhage, and subdural and intracerebral hematoma. A 52-year-old man was admitted to the neurosurgery clinic with severe headache, retro-orbital pain, and blurred vision. Lumbar puncture demonstrated that the CSF opening pressure was 32 cm H _2_ O. A nonprogrammable LP shunt with two distal slit valves was inserted. Shortly after the surgery, his condition deteriorated and he became comatose. Immediate computed tomography scan revealed cerebellar hemorrhage and acute hydrocephalus. Development of remote cerebellar hemorrhage following LP shunt is rare. We discuss this rare event and the applicable literature.

Idiopathic intracranial hypertension (IIH) is characterized by high intracranial pressure without hydrocephalus or intracranial mass. 1 2 The incidence is 1 to 3/100,000 in the general population and 21/100,000 in the obese population. 3 The pathophysiological causes of rising intracranial pressure are still unknown. One of the theories is decreased absorption due to increased cerebral venous pressure. 1 The presence of transverse sinus stenosis is radiologically shown in a large number of patients with IIH. However, it is still not clear whether this finding increases intracranial pressure. 4

The most common symptoms were headache (92%) and blurred vision (35 to 60%). Approximately 67% of these patients had headaches refractory to medical treatment, and progressive optic neuropathy was identified in 52% of the patients. 2 5 Treatment of the IIH depends on the variety of the symptoms and associated clinical findings. If the patient only has headache, medical treatment including diuretics (acetazolamide, topiramate) and analgesics is appropriate. If the patient has headache with accompanying loss of the vision, surgical treatment including optic nerve fenestration and placement of ventriculoperitoneal (VP) and lumboperitoneal (LP) shunts should be more appropriate. 2 6 7 Rarely, steroids are recommended in cases of radiologic transverse sinus stenosis and obesity until surgical treatment can be applied. 4 5 8

For decreasing intracranial pressure, cerebrospinal fluid (CSF) LP shunt is widely used for the surgical management, although it carries complications such as shunt migration, venous sinus thrombosis, subarachnoid hemorrhage, and subdural and intracerebral hematoma. 9 10 11 12 13 We present a case of cerebellar hemorrhage after LP shunt insertion, which resulted in rapid deterioration and coma shortly after surgery. Development of remote cerebellar hemorrhage following LP shunt is a rare complication. We also provide a presumptive biomechanism of intracerebral hematoma due to LP shunt insertion.

## Case Report

**Fig. 1:**
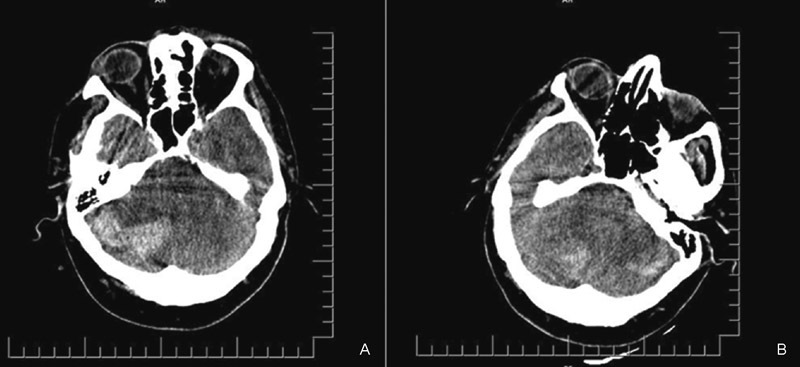
Postoperative cranial noncontrast computed tomography axial section. (A) Hyperdense lesion showing right cerebellar hemorrhage with moderate mass effect. (B) Bilateral cerebellar hemorrhage.

**Fig. 2:**
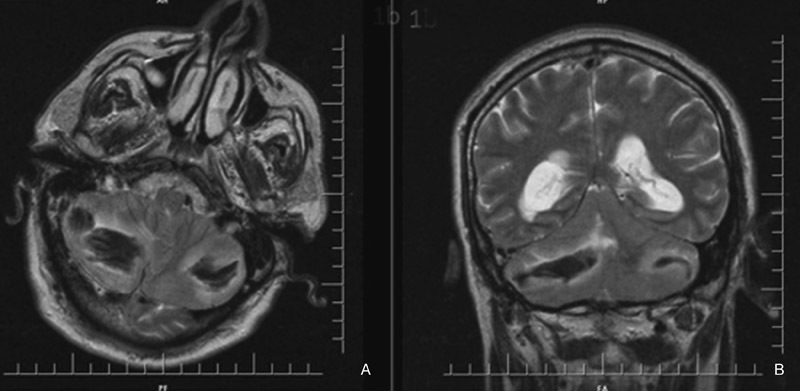
Postoperative T2-weighted cranial magnetic resonance imaging. (A) Axial section and (B) coronal section showing bilateral cerebellar hemorrhage with fourth ventricle compression.

A 52-year-old man was admitted to the neurosurgery clinic with severe headache, retro-orbital pain, and blurred vision. His headache got worse over the next 3 days. He did not have a remarkable history of using any drugs such as steroids, tetracycline, nitrofurantoin, or vitamins. His physical examination was normal, and his body mass index was 40. Neurologic examination was normal but ophthalmologic examination by fundoscopy revealed bilateral papilledema with tortuous vessels. Ophthalmology consultation ruled out other causes of papilledema. Lumbar puncture demonstrated that the CSF opening pressure was 32 cm H _2_ O. Laboratory evaluations of blood and CSF were within normal limits. Brain magnetic resonance imaging (MRI) and magnetic resonance venography revealed normal parenchymal and orbital findings and no dural sinus thrombosis. He was started on acetazolamide (3 × 250 mg, orally) and furosemide (2 × 40 mg, orally) for 4 weeks. However, his symptoms partially improved with serial lumbar punctures. Therefore, a nonprogrammable LP shunt with two distal slit valves (Phoenix Biomedical Corp., Valley Forge, Pennsylvania, United States) was inserted. There was no perioperative complication, and the patient was mobilized 24 hours after surgery. Shortly after the mobilization, his condition deteriorated and he became comatose. Glasgow Coma Scale score was 6/15. Immediate computed tomography scan revealed a remote cerebellar hemorrhage and acute hydrocephalus due to fourth ventricular compression ( Fig. 1 ). The patient was intubated and transferred to the intensive care unit. The LP shunt was obliterated and external ventricular drainage was inserted for hydrocephalus. Cranial MRI and magnetic resonance angiography revealed no aneurysm or arteriovenous malformation as a cause of cerebellar hemorrhage ( Fig. 2 ). He died 9 days after the second surgery.

## Discussion

IIH is characterized by increased intracranial pressure without hydrocephalus or mass lesion and with normal CSF composition. The maximum available medical treatment, such as steroids, acetazolamide, and serial lumbar punctures, should be administered as first-line therapy before placing a shunt. 2 7 Rosenberg et al reported that complications are common and limit the effectiveness of all kind of shunts in procedures for IIH. In their series, 37 patients had a total of 73 LP shunts and 9 VP shunts. 9 10 Only 14 patients remained “cured” after a single surgical procedure. The average time between shunt insertion and shunt replacement was 9 months, although 64% of shunts needed revision within 6 months. Shunt failure (55%) and headaches due to intracranial hypotension (21%) were the most common causes for revision. 10 LP shunts are also associated with a lower complication rate than VP shunts in the treatment of IIH. 11

Although placement of an LP shunt is an effective and relatively safe procedure, a variety of complications associated with LP shunt have been reported in the literature; these include malfunction requiring revision, infection, and migration or fracture of catheter. Some rare complications have also been reported, including radiculopathy, foraminal migration of shunt, tonsillar herniation, spinal deformities, subarachnoid hemorrhage, and acute subdural hematoma. 9 10 11 12 Development of intracerebral hemorrhage following LP shunt is a quite rare complication, and only two cases have been reported in the literature. 10 13 As stated by Turkoglu et al, 11 the potential pathogenesis is overdrainage, but lack of any subdural effusion or blood argues against this possibility. Lumbar CSF drainage may result in a reduction of CSF volume with a related lowering in intracranial pressure. This may eventually increase the transmural pressure gradient of the vessels, leading to a secondary wall stress rupture. Overdrainage of the CSF during the operation may lead to intracerebral hemorrhage. However, cerebellar hemorrhage associated with LP shunt insertion has not been reported in the English language literature. Cerebellar hemorrhage mostly develops remotely from the surgical site and especially occurs after spinal surgeries and supratentorial transcranial surgery due to intracranial hypotension related to a high amount of CSF drainage. 14 15 Drainage of CSF stretches the bridging veins in the posterior fossa, causing temporary occlusion, and increases intraparenchymal venous pressure, which mostly causes venous hemorrhage. 14 15 16 The zebra sign was identified by Brockmann et al as a bleeding pattern of cerebellum following CSF loss. 17 However, in our case blood had already accumulated homogenously in the posterior fossa at the time of brain imaging ( Fig. 1 ).

The LP shunt used in our case was not a programmable shunt. However, it had a slit valve at the distal end, and excessive drainage of CSF did not occur during the operation. After 24 hours, followed by mobilizing the patient, neurologic deterioration and sudden unconsciousness occurred. These findings suggest that an LP shunt could cause excessive CSF drainage depending on the position. Lumbar CSF drainage reduces CSF volume with a related lowering in intracranial pressure. This may eventually increase traction in the posterior fossa bridging veins and may cause remote cerebellar hemorrhage.

In conclusion, although remote cerebellar hemorrhage following an LP shunt procedure is not common, this complication should be remembered when neurologic deterioration occurs after LP shunt placement.
